# A cross-source, system-agnostic solution for clinical data review

**DOI:** 10.1093/database/baz017

**Published:** 2019-02-18

**Authors:** Michael A Farnum, Mathangi Ashok, Daniel Kowalski, Fang Du, Lalit Mohanty, Paul Konstant, Joseph Ciervo, Victor S Lobanov, Dimitris K Agrafiotis

**Affiliations:** Covance, the Drug Development Division of LabCorp, 210 Carnegie Center, Princeton, NJ, USA

## Abstract

Assembly of complete and error-free clinical trial data sets for statistical analysis and regulatory submission requires extensive effort and communication among investigational sites, central laboratories, pharmaceutical sponsors, contract research organizations and other entities. Traditionally, this data is captured, cleaned and reconciled through multiple disjointed systems and processes, which is resource intensive and error prone. Here, we introduce a new system for clinical data review that helps data managers identify missing, erroneous and inconsistent data and manage queries in a unified, system-agnostic and efficient way. Our solution enables timely and integrated access to all study data regardless of source, facilitates the review of validation and discrepancy checks and the management of the resulting queries, tracks the status of page review, verification and locking activities, monitors subject data cleanliness and readiness for database lock and provides extensive configuration options to meet any study’s needs, automation for regular updates and fit-for-purpose user interfaces for global oversight and problem detection.

## Introduction

The goal of clinical trials is to demonstrate the efficacy, safety and comparative effectiveness of new investigational treatments or existing therapies that warrant further study. Data management plays a crucial role in this process, ensuring that the data collected is complete, accurate and delivered in a timely manner for analysis, submission and disclosure (1). That process involves many different groups, such as clinical investigators and site staff, patients, laboratory and imaging vendors, device and technology companies and more, entering data into a multitude ofdisparate systems. Of central importance is the electronic data capture (EDC) system used to capture patient observations at investigational sites. That data undergo a significant amount of scrutiny, from ensuring that site personnel enter the information in a timely and complete manner, to supporting queries and other forms of communication to address concerns for accuracy and consistency, to facilitating multiple rounds of review, particularly for verification of correct transcription from the original source. To aid in this process, the data are inspected through a number of programmed checks, which fall into two categories: (i) those implemented and managed within a particular source system (typically the EDC) and (ii) those implemented and managed outside of any individual source. The latter usually require data to be combined from multiple sources and are implemented in SAS, R or Microsoft Excel, as they may require fairly complex logic. In both cases, the output of these checks is reviewed by data managers who delegate any suspect data to the appropriate parties for verification and correction.

Managing this process is very inefficient and error prone. First, as non-EDC (laboratory, biomarker, imaging, patient-reported, etc.) data account for the majority of the information collected during a clinical trial, queries against those systems or vendors involve a different workflow from that used in the EDC (often relying on emails or telephone calls.) Second, whereas the EDC checks are typically implemented by the data managers who set up the electronic case report forms, the more complex checks and discrepancy logic are implemented by statistical programmers.

One approach to addressing these problems is to bring all the data into the EDC and implement both simple and complex checks within that platform. While this may simplify code management and reuse, it is constrained by the performance and scalability of today’s EDC systems, which were developed many years ago and designed for much smaller volumes of data. More critically, this solution increases the EDC setup time protracting its go-live date and does not solve the communication problem with external vendors who do not use an EDC to capture their data, thus complicating query management and resolution.

A second approach, and the one adopted here, is to integrate all clinical trial data into a data warehouse and use that to drive data management activities. This approach is best exemplified by Oracle’s Data Management Workbench (DMW), (2) which relies on Oracle’s Life Sciences Hub (LSH) (3) as the underlying data repository. DMW offers a number of important features, including integrated access to clinical trial data, configurable data management workflows, extensive query, discrepancy and library management capabilities, tight integration with Oracle’s InForm EDC, (4) and more. While DMW does, in principle, enable integration with other EDC systems, we are not aware of any sponsor who has actually reduced this configuration to practice. Perhaps the greatest limitation of DMW is its reliance on LSH, a clinical data warehouse that consists of thousands of tables, which makes it challenging to deploy and maintain.

Recently, we introduced a comprehensive application suite, known as Xcellerate, that uses advanced data integration, analytics and visualization capabilities to improve patient safety, data quality and protocol compliance throughout the clinical development process and enable greater transparency and oversight of study conduct and performance (5, 6). The system consists of a number of end-user applications connected to a clinical data repository that supports near-real-time acquisition, mapping and integration of clinical trial data from any germane source (7, 8). The solution described herein leverages this clinical data repository to streamline and automate the process of delivering high-quality study data and associated metrics for downstream use. It consists of the following four web-based applications: (i) the ‘Discrepancy Manager’, which facilitates review of programmed discrepancy check output and bulk handling of the resulting queries to sites and third-party data vendors; (ii) the ‘Page Tracker’, which facilitates reporting of missing EDC pages and the backlog of outstanding work for page review, verification and locking; (iii) the ‘Query Tracker’, which provides reporting and analytics for query management and root cause analysis; and (iv) the ‘Subject Tracker’, which tracks subject data cleanliness and readiness for database lock and helps data management teams manage outstanding work.

## Methods

### Page Tracker

Page Tracker facilitates the execution and reporting of data entry into the EDC. One of the core functionalities of the tool is the ability to define the visit, page entry and source data verification (SDV) schedules, and the rules that determine which data should be expected when and under what conditions. We use the following three types of rules:

(i) Visit date rules, which determine the time when forms related to a specific study visit should be expected. These rules allow the use of multiple baselines and reference points to support complex study designs that are becoming increasingly prevalent in drug development, such as oncology treatment cycles that may have different schedules for subjects assigned to different study arms.

(ii) Page entry rules, which determine data entry conditions for EDC pages. These rules are used to define the subset of patients and/or pages applicable to a given visit and may utilize any type of data coming from either EDC or non-EDC sources to set the appropriate flag. The most common example is to specify pages applicable to only one gender.

(iii) SDV rules, which define conditions for source data verification. These conditions are originally defined in the EDC system when the study is set up, and we use the EDC metadata to populate the corresponding entries in the SDV schedule.

**Figure 1:**
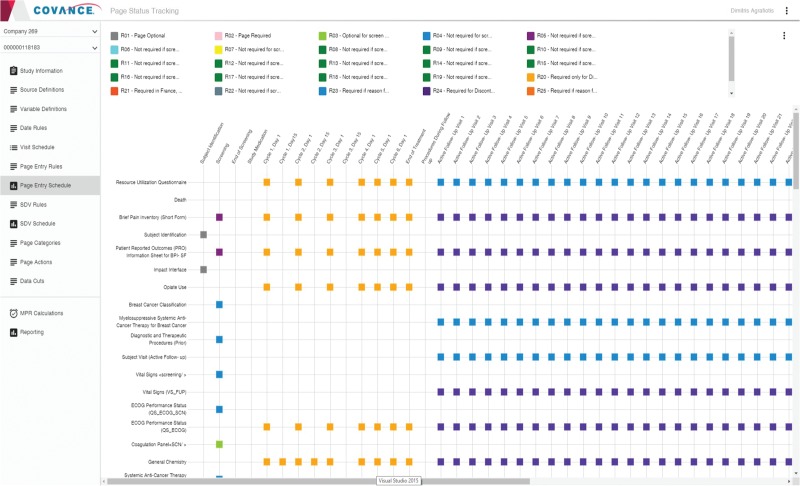
Page entry schedule editor in Page Tracker.

**Figure 2:**
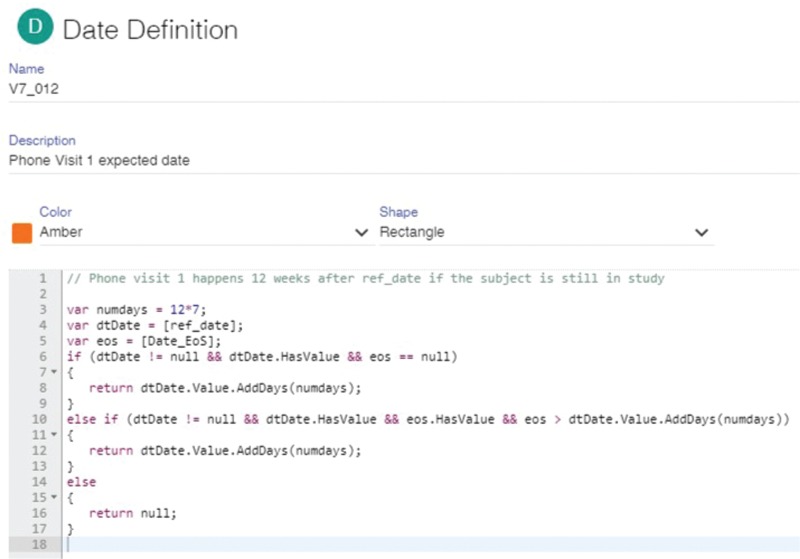
An illustrative visit date rule defined in the Page Tracker. This particular rule specifies that phone visit 1 must occur 12 weeks after the reference date if the subject is still in the study. The rule utilizes two dates: the reference date for this specific visit and the end-of-study (EOS) date. If the reference date is not null and the EOS date is null, phone visit 1 is expected 12 weeks after the reference date. If EOS is not null but happens after the expected phone visit date, the rule returns this expected date, otherwise it returns null to indicate that a phone visit is not expected for this subject because the study has already finished.

Once all the rules are defined, the visit, page entry and SDV schedules are initialized from the EDC metadata and refined interactively using the Page Tracker user interface (UI). In addition, page categories and page actions are specified to help review the status of data cleanliness and completeness (i.e. show how many expected pages are pending, completed, verified, reviewed, frozen and locked). The page entry scheduler is illustrated in Figure 1 and a representative visit date rule is shown in Figure 2.

**Figure 3:**
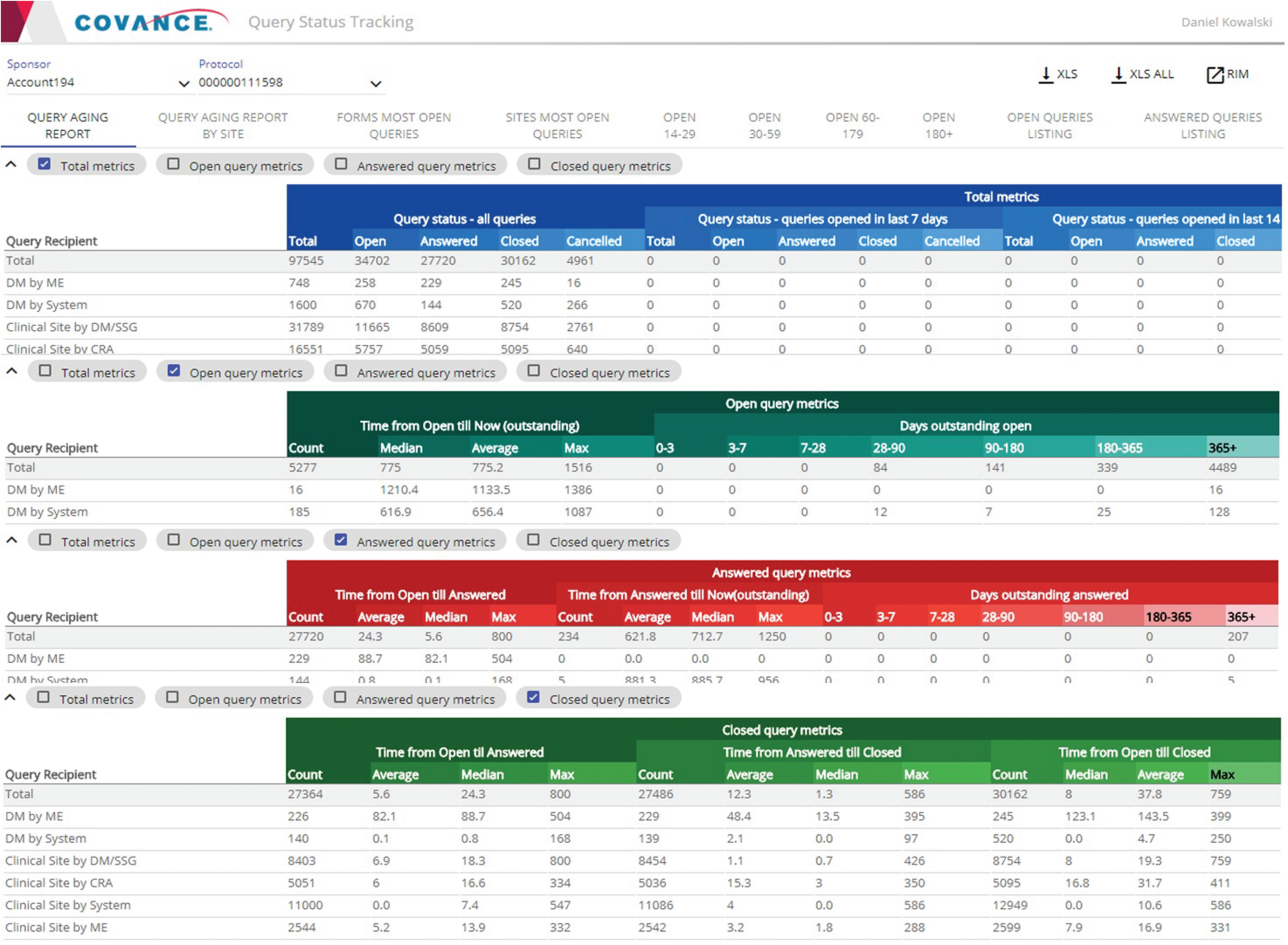
Representative screenshots of the Query Tracker.

**Figure 4:**
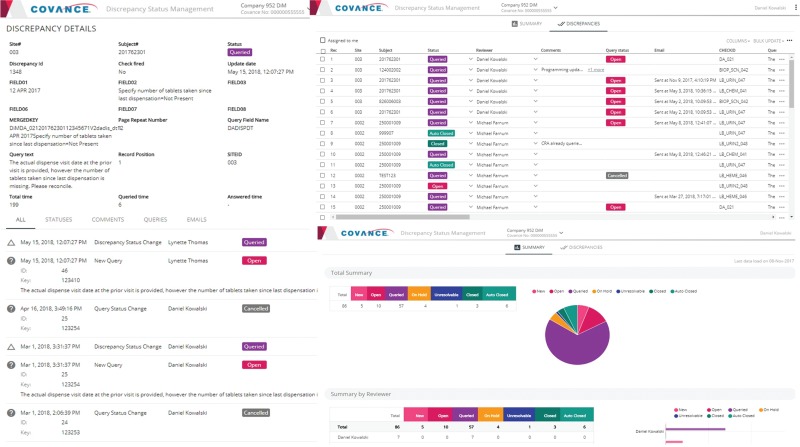
Representative screenshots of the Discrepancy Manager.

By applying these predefined rules, schedules, categories and actions on the latest snapshot of the data in our clinical data repository [which is refreshed nightly from the source systems though automated extraction, transformation and loading (ETL) pipelines], the system tracks the amount of completed and remaining work and allows data managers and clinical research associates (CRAs) to identify sites, subjects and forms with pending actions. The actual calculations can be scheduled to run automatically at any desired frequency or triggered manually through the UI at any time. When completed, the results (including form, page, site, subject and action summaries) become available through the UI and the appropriate personnel are notified by email.

The tool also allows users to define and track arbitrary cohorts that may be of special interest or priority at some point during the trial. These subsets, commonly referred to as data cuts, may be limited to specific subjects, visits, dates or any combination thereof.

### Query Tracker

Queries are an essential component of any modern EDC, indicating potential issues in the data collected. The number of queries and the elapsed time before a query is acknowledged and closed are important determinants of study and site risk and performance (9). Inadequate visibility into such metrics can result in poor data quality and delayed deliverables.

The Query Tracker (Figure 3) is an application that provides query metrics (listings, counts and percentages, cycle times) organized by site, subject, form, marking group (system, user, etc.), status (open, answered, closed), age (days open) and recipient. The tool provides drill-down to individual query details, extensive searching, sorting, filtering, exporting capabilities and direct integration with the Xcellerate Risk and Issue Management (RIM) system (10) to escalate any problematic findings into issues and actions for mitigation.

Besides keeping track of the workload, these query metrics can help users identify systemic problems and trends, and possible solutions. For example, looking at the total counts and percentages of open queries per page or form allows users to identify potential misfiring checks due to logic or programming errors, issues with EDC page design or gaps in site training. Likewise, an excessive number of open queries per site or subject, a high ratio of open queries to pages entered or excessive aging of queries at a given site may be a sign of poor site performance. While spikes should be expected due to the periodic nature of data review, sustained trends over a prolonged period of time could indicate quality issues and higher site risk.

### Discrepancy Manager

While EDC edit checks can work well for simple data entry issues, more advanced checks are still needed to ensure overall data quality. These include data reconciliations, where information is compared across different data sources (e.g. EDC vs. Interactive Voice Recognition System (IVRS), central laboratory or ePRO), programmable protocol deviations and other complex checks that are difficult or impossible to program within the EDC itself. These checks are typically implemented and executed in external software packages like SAS, and their output is delivered to data managers for review and follow up. As this output usually comes in the form of Excel listings, tracking and updating the discrepancy list is very labor intensive and error prone.

The Discrepancy Manager (Figure 4) is a web-based, multi-user, collaborative tool that can import discrepancies identified through any external application or process and manage their entire lifecycle within the system. The tool automatically recognizes any new and updated records and utilizes the web services application programming interfaces (API) that are available in most leading EDCs to open, close or cancel queries, either individually or in bulk, directly from its UI. This obviates the need for data managers to navigate to a specific study, site, subject, visit, form and field within the EDC and manage each query individually, introducing tremendous efficiency in the query management process. For vendors who do not provide query management and API integration capabilities (laboratories, biomarkers, imaging, etc.), the system can send and receive emails with attachments containing the selected discrepancies and capture the entire sequence of every incoming and outgoing email communication for every query or discrepancy originating within the system. The system provides a full audit trail of every action taken within the tool and makes it readily available for inspection during an audit.

### Subject Tracker

**Figure 5:**
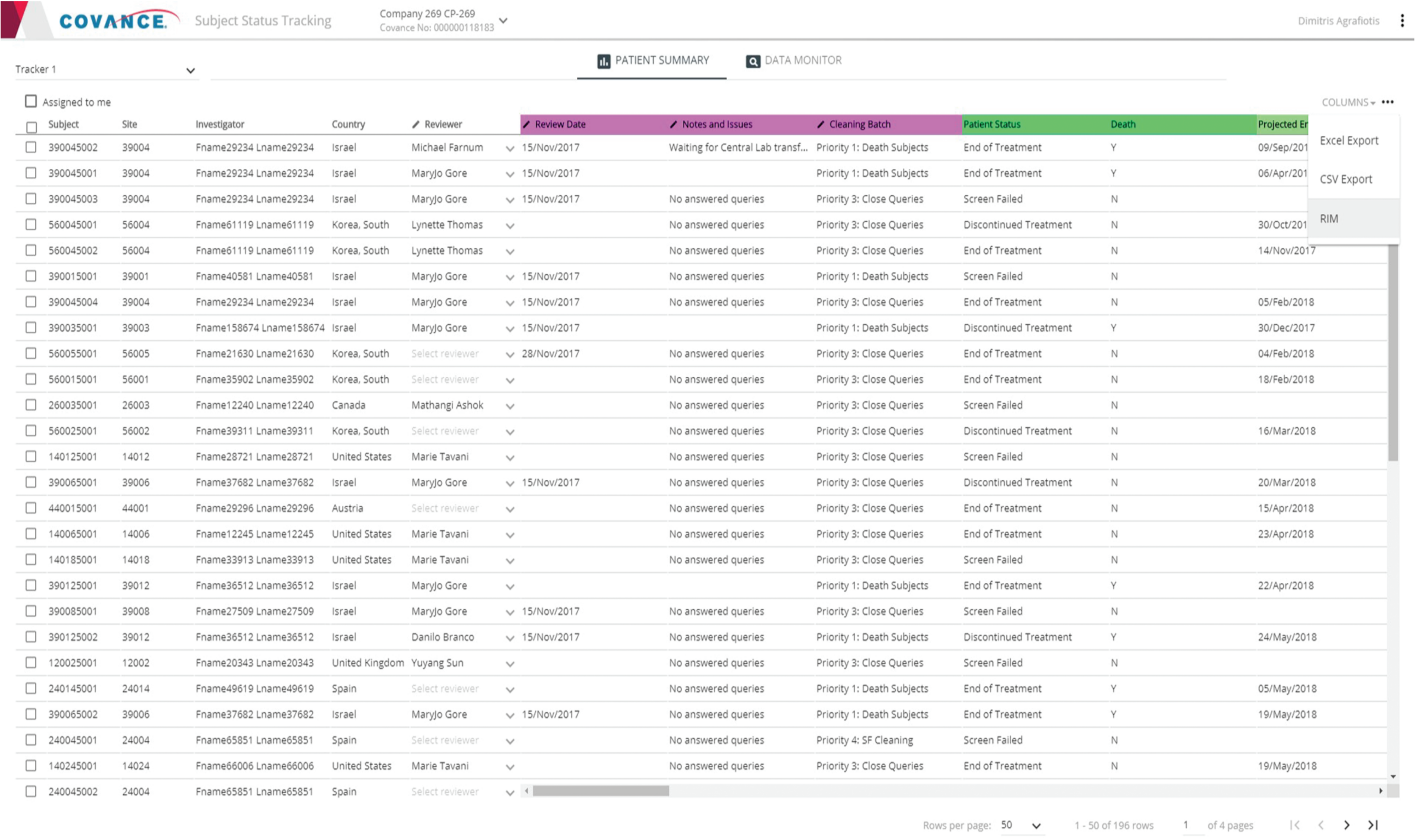
Representative screenshot of the Subject Tracker.

Finally, the Subject Tracker (Figure 5) provides comprehensive patient-level summaries of data cleanliness and completeness to help assess readiness for database lock. The information displayed includes basic milestones and target dates (treatment allocation date, projected end of treatment date, last visit date, etc.) and aggregate statistics on page entry, review, verification, freezing and locking activities, queries and reconciliation activities, etc.

### Architecture

**Figure 6:**
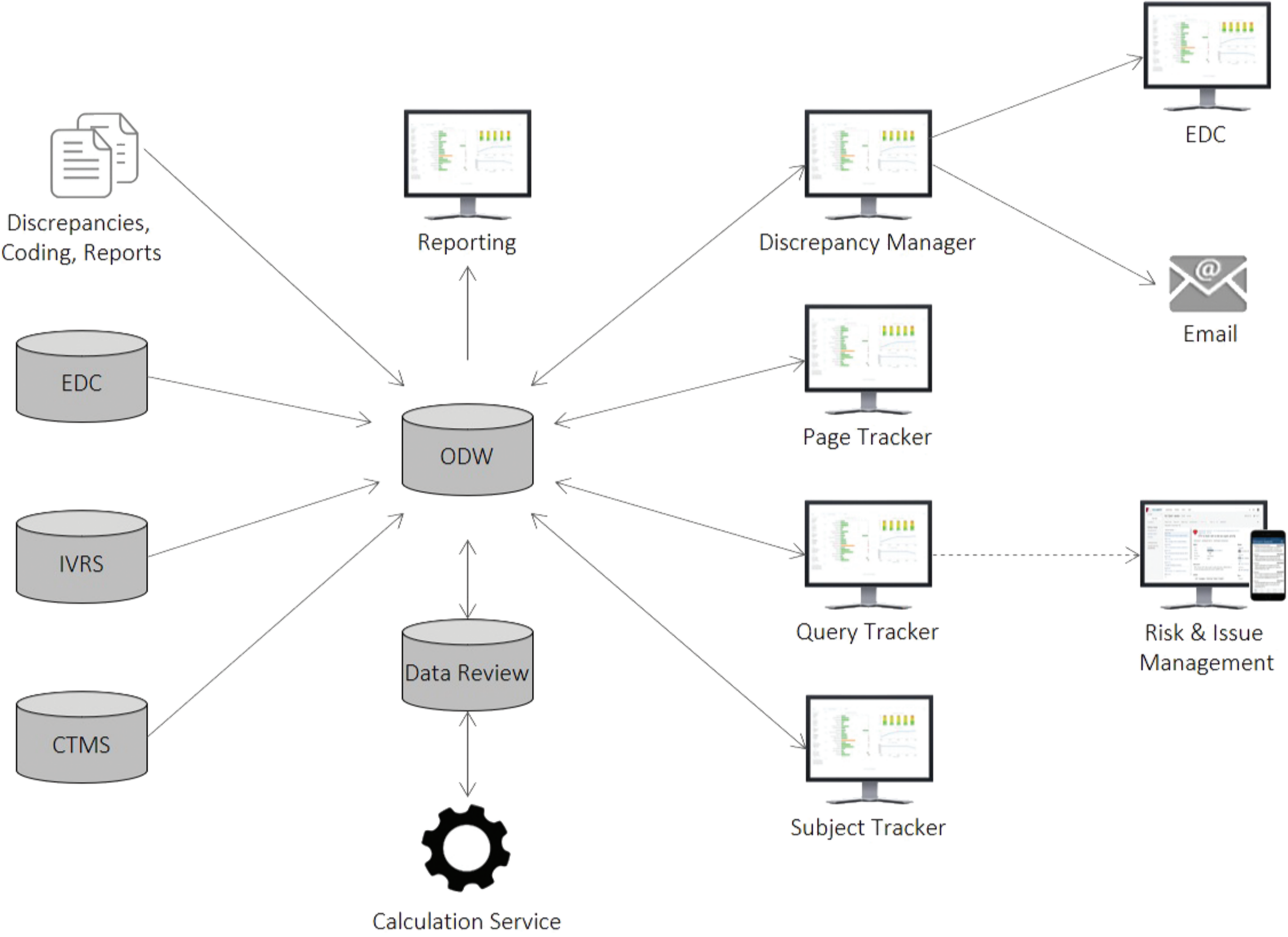
Xcellerate Data Review architecture and data flow.

Our solution consists of several components integrated into a complete, end-to-end system, as illustrated in Figure 6. All source data are initially staged, transformed and loaded into the Xcellerate Operational Data Warehouse (ODW) (7). Protocol, site, subject, query and page data are loaded from the Clinical Trial Management System (CTMS), Interactive Voice/Web Recognition System (IXRS) and EDC through automated ETL processes, whereas other data, such as SAS discrepancy listings and EDC coding and report data that is not available through the EDC APIs, are loaded using a custom file loader. Once the data is loaded, all necessary calculations, including those for page entry and SDV status, are performed by a scheduling engine based on study-specific settings and rules configured through the application UIs during study setup. The results are stored in the data review database (DRDB) and fed back into ODW for reporting purposes and are immediately accessible and actionable by central monitors through the application UIs.

Direct integrations with the EDC and RIM systems allow users to raise queries and generate issues and actions as they review the results. The Query Tracker integrates with RIM through a software development kit that utilizes RIM’s Representational State Transfer (REST) APIs to allow data managers to create issues and actions that become immediately available in the RIM (10) and CRA Dashboard (7) UIs to enable communication, collaboration and rapid follow-up. The Discrepancy Manager integrates with any EDC, including Rave (11) and InForm, (4) using available web services APIs in a loosely coupled, platform-agnostic manner, which allows users to raise queries in the EDC in real time. Responses received in the EDC are loaded onto ODW using the EDC ODM API and from there onto the DRDB using the ETL process mentioned above. For vendors who do not provide query management and API integration capabilities, the system allows for email creation and ingestion through a standard email server. This enables the system to send and receive emails with attachments containing the selected discrepancies and capture the entire sequence of every incoming and outgoing email communication in DRDB.

The physical architecture consists of multiple independent services, implemented using a standard three-tier architecture with shared data stores. The ODW and DRDB are implemented on SQL Server 2014 (12). Data integration is implemented through a combination of Informatica ETL (13) and a custom .Net file loader. The application layer, including the RESTful APIs and calculation service, are also implemented in .Net. The application layer uses Entity Framework (14), Dapper MicroORM (15) and ADO.Net (16) to access the data layer and the open-source Hangfire framework (17) to provide background job processing for the calculation engine. The presentation layer consists of browser-based, single-page web applications based on the Angular 2 JavaScript framework (18) along with additional third-party UI control libraries. Visualizations in all applications were implemented using the Xcellerate JavaScript visualization library that was originally developed for the Xcellerate Medical Review application (5).

## Results

The problem of missing data in clinical trials has been widely discussed (19). Although there has been extensive research on the use of imputation, weighting, slope estimation and other methods to reduce the impact of missing data (minimize bias and preserve the statistical power of the study), very little has been published on its prevention (20). As stated in EMA’s Guideline on Missing Data in Confirmatory Clinical Trials: ‘it is extremely important to avoid the presence of unobserved measurements as much as possible, by favouring designs that minimize this problem, as well as strengthening data collection regardless of the patient’s adherence to the protocol’ (21). Efficient review and management of data queries triggered by SDV, programmed edit checks, header reconciliation and database lock activities can help minimize data quality issues, cost overruns and delays. However, to improve the prevention of these issues, one needs to identify their root cause. A comprehensive data management approach should include ongoing analysis and mitigation of the most frequent sources of queries and missing data, both from an operational (which sites/subjects) and a quality-by-design (which forms/procedures) perspective.

The system presented herein includes a combined database of subject visits from study protocol and IXRS, pages and queries sourced from EDC, discrepancies from data checks programmed in SAS or other external tools and protocol deviations from CTMS or other issue management systems. A purpose-built graphical UI allows data managers to identify sites, EDC forms or study procedures responsible for higher query rates, discrepancies and protocol deviations. The interface also flags sites that fall behind on data entry and query resolution. Further, integration with the ODW and EDC allows visual and interactive review of identified data discrepancies with an integrated capability to generate data queries to sites and any resulting findings are communicated to central and site monitoring staff for action assignment via our RIM system.

Our solution helps reduce avoidable data errors and loss by providing timely and integrated access to all clinical trial data, eliminating unnecessary and duplicative work, enabling communication, collaboration and oversight, facilitating reporting and trending and enhancing the overall user experience. Furthermore, users can easily determine which issues are most prevalent and have the greatest impact on study quality and performance. By adopting adequate issue management measures to identified problems, risk-based corrective and preventive actions can be undertaken. This approach is fully in line with recent ICH E6 R2 guidance that states: ‘The sponsor should develop a systematic, prioritized, risk-based approach to monitoring clinical trials. […] Review, that may include statistical analyses, of accumulating data from centralized monitoring can be used to: (a) identify missing data, inconsistent data, data outliers, unexpected lack of variability and protocol deviations; (b) examine data trends such as the range, consistency and variability of data within and across sites; (c) evaluate for systematic or significant errors in data collection and reporting at a site or across sites, or potential data manipulation or data integrity problems; (d) analyze site characteristics and performance metrics; (e) select sites and/or processes for targeted on-site monitoring’ (22).

Akin to personal finance software that helps consumers manage all their accounts from a single site, our solution creates a layer of abstraction that allows seamless connectivity to the source systems, provides comprehensive and up-to-date views of data cleanliness and completeness, enables bulk management of queries, offers traceable database persistence and documentation of every activity, supports multiple modes of communication and enables continuous data cleansing and reconciliation that reduces site and study risk and speeds up database lock.

By utilizing data integrated in ODW, complex, cross-vendor rules may be created to define conditions for missing data and simplify reconciliation across multiple data sources. Automatic detection of new and updated records minimizes redundant work and helps users focus on the latest issues. Queries can be managed individually or in bulk in a system- and vendor-agnostic way, either through direct API integration with the EDC or through email for vendors who do not support API endpoints. The entire history of every query and every discrepancy is tracked consistently regardless of source and is readily available for inspection during an audit. A comprehensive, up-to-date view of the status and quality of the data is readily available through the master subject tracker to help assess readiness for database lock. Finally, the calculations can be run on a preset schedule or at any time upon request throughout the life of the trial and alerts and notifications drive immediate action when predefined thresholds on inactivity or backlog are exceeded.

## Discussion

Owing to the heavily regulated nature of the pharmaceutical industry and the protracted length of clinical trials, introduction of new clinical systems requires time and careful planning, and legacy tools tend to linger on long after their functionality has been outdated by newer technologies (23). The Discrepancy Manager, Page Tracker and Subject Tracker were released to production in November 2017 and the Query Tracker in July 2018. As of this writing, the tools are being used in 16 active studies (still relatively early in their course) and are being configured for 15 additional ones. While the technology is too early on its adoption curve and quantitative evidence of impact is still lacking, the feedback received from the pilot teams has been very encouraging, suggesting significant improvements in usability, productivity, quality and compliance, as well as in employee engagement and retention in an area where turnover has been historically high, partly due to the lack of modern tools to reduce repetitive, manual work. Performance metrics along all of these dimensions will be provided in a subsequent publication.

It is important to make a distinction between ‘data review’ and ‘medical review’. The tools presented here address operational aspects of clinical data quality, which are distinct from, and complementary to, the review performed by study physicians. Data review allows data managers to identify and correct missing, erroneous and inconsistent data and ensure that the data sets submitted for statistical analysis are complete and error free. Medical review allows physicians to assess patient safety, protocol deviations and other clinical issues that may degrade trial integrity and involves periodic assessment of patient demographics, adverse events, medical history, concomitant medications, laboratory results, clinical endpoints, protocol deviations, disposition events and many other types of patient data collected during the course of study. Our tool for medical review will be presented in a subsequent publication (24).

## Conclusion

We have presented an integrated solution for clinical data review that helps data managers identify missing, erroneous and inconsistent data, manage queries and organize their workload in a unified, system-agnostic and efficient way. The system centralizes all data review and cleansing activities under a common UI, enabled by an operational data warehouse that integrates in near real time all relevant clinical data collected during the course of the trial, communicates seamlessly with the source systems and maintains a complete audit trail of every change in the underlying data. Planned enhancements include a new integrated environment that will greatly simplify the development, maintenance and reuse of complex edit checks, programmable deviations, reconciliations and other data validation and quality checks and additional automation to facilitate database lock and the generation of submission-ready Standard Data Tabulation Model (SDTM) data sets.
